# Development of a multiplex polymerase chain reaction technique for detection and discrimination of *Eimeria* spp. in cattle in Indonesia

**DOI:** 10.14202/vetworld.2022.975-980

**Published:** 2022-04-18

**Authors:** Fitrine Ekawasti, Raden Wisnu Nurcahyo, Mukh Fajar Nashrulloh, Dwi Priyowidodo, Joko Prastowo

**Affiliations:** 1 Indonesia Research Center for Veterinary Science, National Research and Innovation Agency, Bogor, 16114, Indonesia; 2 Department of Parasitology, Faculty of Veterinary Medicine, Universitas Gadjah Mada, Yogyakarta, 55281, Indonesia; 3 Applied Zoology Research Center, National Research and Innovation Agency, Bogor, 16911, Indonesia

**Keywords:** bovine, diagnostic, eimeriosis, multiplex, nested, Sulawesi island

## Abstract

**Background and Aim:**

Bovine eimeriosis is a disease caused by apicomplexan parasites of the genus *Eimeria*. It is one of the most important and widespread bovine illnesses in the world. Some of the identified species of bovine eimeriosis have morphologically similar oocysts that are difficult to differentiate. For the identification of particular *Eimeria* spp., diagnostic laboratories are increasingly turning to DNA-based technology. This study aims to develop a multiplex polymerase chain reaction (mPCR) technique based on the internal transcribed spacer-1 (ITS-1) gene for the simultaneous identification of pathogenic *Eimeria* spp. in cattle from Sulawesi Island, Indonesia.

**Materials and Methods:**

Genomic DNA was extracted by the DNAzol reagent from the purified *Eimeria* oocysts. Species-specific primers targeting the ITS-1 region were used to amplify the distinct *Eimeria* spp.

**Results:**

Using PCR ITS-1, this study showed that 36 of 120 fecal samples (30%) were infected by *Eimeria* spp. The multiplex PCR assay allowed for the simultaneous identification of six major *Eimeria* spp. in a single-tube reaction. The proportion of mixed *Eimeria* spp. infections was 100% (36/36). The maximum number of *Eimeria* spp. was five, and the minimum number was two.

**Conclusion:**

Identification of six pathogenic *Eimeria* spp. in cattle was successfully carried out by nested multiplex PCR using ITS-1 gene. In the future, a procedure to detect pathogenic *Eimeria* spp. in one tube reaction will offer economical and save diagnostic time.

## Introduction

Bovine eimeriosis is a disease caused by apicomplexan parasites of the genus *Eimeria*. It is one of the most important and widespread bovine illnesses globally [1]. It is regarded as one of the five most commercially significant illnesses in the cattle industry. Bovine eimeriosis frequently has an impact on the health of the host, resulting in reduced meat output in beef cattle [2,3]. *Eimeria* spp. are completely host-specific, with over 20 spp. of *Eimeria* found in cattle [4]. Coccidiosis illnesses are frequently asymptomatic, although they can result in unexpected death in animals. Because the morphological features of *Eimeria* spp. are comparable in form and size between species, the diagnostic approach that is still employed today, which is based on morphology, should not be utilized in identifying *Eimeria* spp. To effectively control coccidiosis, a suitable *Eimeria* diagnostic approach is required. Because they cause the clinical indications listed above, *Eimeria bovis*, *Eimeria zuernii, Eimeria alabamensis*, *Eimeria auburnensis*, *Eimeria cylindrica*, and *Eimeria ellipsoidalis* are assumed to be pathogenic [5]. *E. bovis* and *E. zuernii* have a significant impact because of their high pathogenicity, particularly on mortality in calves under 1 year of age. These pathogens are frequently associated with diarrheic feces containing blood, fibrin, and intestinal tissues, causing morbidity and mortality by disrupting intestinal absorption [6,7].

Indonesia’s beef cattle population was predicted to be approximately 16.5 million in 2018 [8]. Beef cattle are one of the most important sources of meat in Indonesia. Sulawesi is a key area for the Indonesian government’s beef cattle program to increase the country’s food supply and fulfill the demand for meat [9]. The Indonesian Ministry of Agriculture has begun drafting strategic plans for food self-sufficiency projects [8]. The efficacy of these approaches is thought to be connected to gastrointestinal parasites, such as bovine eimeriosis, which affect calves. Ekawasti *et al*. [10] reported a species-specific and sensitive polymerase chain reaction (PCR) test for bovine *Eimeria* in Java, Indonesia, using ribosomal internal transcribed spacer-1 (ITS-1). PCR is a more reliable, sensitive, and time-efficient method of diagnosing *Eimeria* [7]. Because it is easy to amplify from tiny quantities of DNA and has a high degree of variation across closely related species, the ITS region is widely employed in molecular phylogeny and taxonomy [10-12].

The development of a simple, rapid, and cost-effective detection method for bovine *Eimeria* is critical to determine the infection rate in the community as well as in individuals. Multiplex PCR detects many targets simultaneously, with less time and cost than regular PCR [13].

This study aims to develop a multiplex PCR assay as a simple and rapid diagnostic technique for simultaneously detecting and discriminating six *Eimeria* spp. in cattle.

## Materials and Methods

### Ethical approval

The study was approved by Research Ethics Committee, Faculty of Veterinary Medicine, University of Gadjah Mada, Indonesia (Nomor: 00032/EC-FKH/Int./2020)

### Study period and location

The study was conducted from November 2020 to April 2021. Fecal samples were collected from four provinces on Sulawesi Island. One fecal sample was obtained from each animal. The weather condition during the sample collection period was the dry season.

### Collection of fecal samples

**Figure-1:**
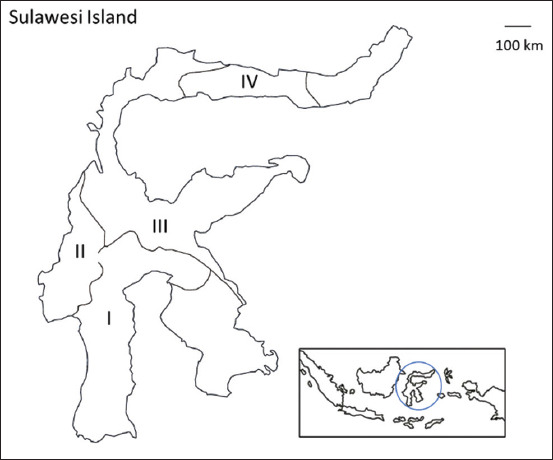
The locations of study area on Sulawesi Island. The numbers in Figure-1 are also used in Table-2. [Source: https://akupetagambar.blogspot. com/2016/02/peta-indonesia-outline.html].

A total of 120 fecal samples from beef cattle were obtained from four provinces on Sulawesi Island, with each study site having more than 2 farms. Figure-1 depicts the sampling locations. Fecal samples were obtained directly from the rectums of beef cattle with the use of examination gloves, preserved in separate plastic bags, and refrigerated at 4°C for laboratory testing. There were no clinical symptoms in any animals when the fecal samples were taken.

**Table 1:** Primer sets used for the polymerase chain reaction [11].

Species	Primer sequences (5-3)		Expected product size (bp)

	Forward	Reverse	
Genus common	gcaaaagtcgtaacacggtttccg	ctgcaattcacaatgcgtatcgc	348-546
*Eimeria bovis*	tcataaaacatcacctccaa	ataattgcgataagggagaca	238
*Eimeria zuernii*	aacatgtttctacccactac	cgataaggaggaggacaac	344
*Eimeria alabamensis*	cattcacacattgttctttcag	gcttccaaactaatgttctg	184
*Eimeria auburnensis*	taaattggtgcgatgaggga	gcaatgagagaaagatttaata	295
*Eimeria cylindrica*	gacatttaaaaaaccgattggt	ggctgcaataagatagacata	304
*Eimeria ellipsoidalis*	caacgtttttccttttcctatca	actgcgatgagagagagcg	148

**Table 2:** Summary of study areas and PCR analysis for *Eimeria* spp.

No.	Province	Number of sample tested	Number of sample positive PCR	PCR analysis*						Notes

				*E. b* 238 bp	*E. z* 344 bp	*E. alab* 184 bp	*E. aubu* 295 bp	*E. cylin* 304 bp	*E. elip* 148bp	
I	South Sulawesi	30	12 (40%)	12	7	5	7	5	4	Mix with two species (2); three species (6); four species (2); five species (2)
II	West Sulawesi	30	8 (26.7%)	4	4	0	3	1	1	Mix with three species (6); four species (2)
III	Central Sulawesi	30	6 (20%)	6	4	0	0	2	4	Mix with two species (2); three species (4)
IV	Gorontalo	30	10 (33.3%)	10	2	2	0	8	0	Mix with two species (8); three species (2)
Total		120	36 (30%)	32 (88.9%)	17 (47.2%)	7 (19.4%)	10 (27.8%)	16 (44.4%)	9 (25%)	

* *E. b*=*Eimeria bovis*, *E. z*=*Eimeria zuernii*, *E. alab*=*Eimeria alabamensis*, *E. aubu*=*Eimeria auburnensis*, *E. cylin*=*Eimeria cylindrica*, *E. elip*=*Eimeria ellipsoidalis*, PCR=Polymerase chain reaction

### Processing of fecal samples

*Eimeria* oocysts were isolated from the feces by the sugar flotation technique [10]. The feces were diluted in distilled water and filtered through gauze. After a 5 min centrifugation at 800× *g*, a sugar solution was added to the sediments, and then distilled water was overlaid and centrifuged at 1200× *g* for 10 min. *Eimeria* oocysts that floated on the surface of the sugar solution were retrieved with a Pasteur pipette and rinsed 3 times with distilled water. Finally, the pure oocysts were kept at 4°C after being redissolved in 1-2 mL of phosphate-buffered saline.

### Isolation of genomic DNA

Only samples containing more than 20 oocysts of each species were used to isolate genomic DNA [10,14]. In brief, 500 μL of pure *Eimeria* oocysts were combined with 500 μL of DNAzol and freeze-thawed 5 times to extract genomic DNA. DNAzol (Molecular Research Center, OH, USA) was used to extract DNA from the purified *Eimeria* oocysts. Purification and concentration of the DNA extracts for DNA quantification were performed using Thermo Scientific NanoDrop Products, and the extracts were stored at −20°C before PCR testing, using the extracted DNA as a template.

### Molecular identification of *Eimeria* spp.

The genetic markers used in this study using internal transcribed spacer-1 (ITS-1). The primers used are presented in Table-1 [11].

### Identification of genus *Eimeria* genomic DNA

PCR amplification was used to determine the presence of genomic DNA from the genus *Eimeria* [11]. In a 25 μL reaction mixture, each reaction comprised 100 ng of templates, 10 μM each of forward and reverse primers, 0.5 μL of MyTaq HS DNA polymerase, and 5 μL of 5X MyTaq Reaction Buffer (Bioline, UK). An initial denaturing phase at 95°C for 3 min was followed by 35 cycles at 95°C for 1 min, 55°C for 1 min, 72°C for 2 min, and a final extension at 72°C for 5 min. At 100 V, electrophoresis with 1.5% agarose (UltraPure™ Agarose product, Invitrogen, US) and SYBR Safe DNA Gel Stain was performed (Thermo Fisher Scientific, Finland). An ultraviolet transilluminator was used to visualize the bands.

### Nested PCR

The nested PCR procedure using ITS-1 primers was standardized for the detection of bovine *Eimeria* spp. [11]. Species-specific primers targeting the ITS-1 region were used to amplify the distinct *Eimeria* spp. [11]. The product of the primary PCR (first, genomic) (1 L in 25 μL of reaction mixture) was used as a template for the nested PCR (second, species specific) with six species-specific primers (*E. bovis*, *E. zuernii*, *E. alabamensis*, *E. auburnensis*, *E. ellipsoidalis*, and *E. cylindrica*) in individual tubes using the same amplification conditions described above (first PCR). Negative, no-template controls were supplied with each experiment, and distilled water was used in place of the template. Gel electrophoresis in 1.5% agarose (UltraPure™ Agarose product, Invitrogen) was used to verify the amplification of certain nested multiplex PCR products.

### Development of multiplex PCR

The nested multiplex PCR using ITS-1 primers identified the six *Eimeria* spp. that infect cattle. The primary PCR product (first, genus *Eimeria* genomic) was used as a template for the nested PCR (second, species-specific), with six primers in a single tube for multiplex PCR to detect species-specific *Eimeria* simultaneously.

In a 25 μL reaction mixture, each reaction comprised 100 ng of templates, 10 μM each of forward and reverse primers (six pairs of primers in the second PCR), 0.5 μL of MyTaq HS DNA polymerase, and 5 μL of 5X MyTaq Reaction Buffer (Bioline). An initial denaturing phase at 95°C for 3 min was followed by 35 cycles at 95°C for 1 min, 55°C for 1 min, 72°C for 2 min, and a final extension at 72°C for 5 min.

## Results

All four provinces of Sulawesi were confirmed to be positive for *Eimeria* spp. in our fecal sample investigations. PCR tests for six *Eimeria* spp. identified the species in 36 of the 120 samples, indicating that *E. bovis* was the most common. Table-2 provides an overview of the findings.

To achieve a common response condition for the six *Eimeria* spp., nested PCR singles (PCRs) were first used to test for separate reactions of each ITS-1-PCR primer pair. We obtained PCR products with a size of 348-546 bp from the first PCR amplification in the common-genus *Eimeria*, then followed by a second PCR (nested PCR).

**Figure-2:**
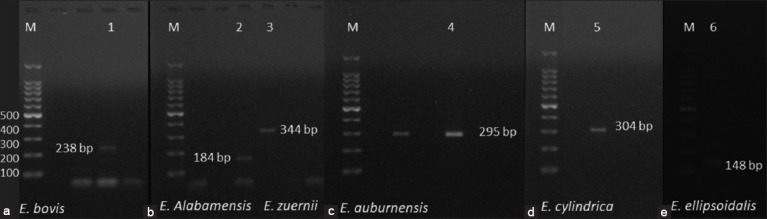
The six individual polymerase chain reactions using purified DNA samples. Lane M, molecular size marker; (a) *Eimeria bovis*, 238 bp; (b) *Eimeria alabamensis*, 184 bp; (c) *Eimeria zuernii*, 344 bp; (d) *Eimeria auburnensis*, 295 bp; (e) *Eimeria cylindrica*, 304 bp; and (f) *Eimeria ellipsoidalis*, 148 bp DNA fragment.

**Figure-3:**
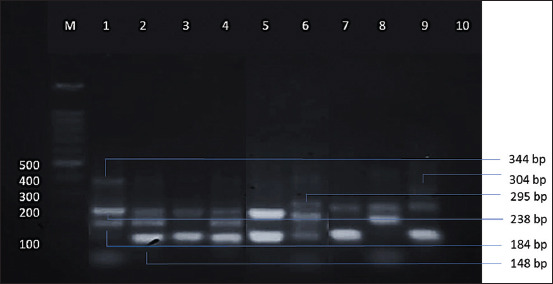
The six primer pairs of Eimeria spp. were tested in the multiplex polymerase chain reaction using purified DNA samples.

PCR products were amplified from the second PCR: 238, 344, 184, 295, 304, and 148 bp for *E. bovis*, *E. zuernii*, *E. alabamensis*, *E. auburnensis*, *E. cylindrica*, and *E. ellipsoidalis*, respectively (Figure-2). The six primer pairs were tested with the multiplex PCR using purified DNA of six species of *Eimeria* (Figure-3). The test allowed for the simultaneous identification of six *Eimeria* spp. in a single-tube reaction, as evidenced by a homogeneous band ladder. Individual PCRs, as well as multiplex PCRs, were used to visualize *E. bovis*, *E. zuernii*, *E. alabamensis*, *E. auburnensis*, *E. cylindrica*, and *E. ellipsoidalis* amplicons (Figure-2 and 3).

The one-tube multiplex PCR detected *E. bovis*, *E. zuernii*, *E. alabamensis*, *E. auburnensis*, *E. cylindrica*, and *E. ellipsoidalis* in 88.9%, 47.2%, 19.4%, 27.8%, 44.4%, and 25% of field samples from Sulawesi Island, respectively (Table-2). All samples (36/36) were infected with more than 1 *Eimeria* spp. Mixed infections with five *Eimeria* spp. were found in 5.5% of samples (2/36), four species in 11.1% (4/36), three species in 50% (18/36), and two species in 33.3% (12/36).

## Discussion

In this study, the ITS-1 site of DNA from several pathogenic *Eimeria* spp. in beef cattle were used to design primers because this is a highly conserved territory among species [11]. PCR tests capable of detecting and distinguishing *Eimeria* spp. have been available for more than 20 years. However, despite their status as the “gold standard” of detection for many diseases, conventional coccidial diagnostics have yet to be replaced [14,15]. The resistance of the oocyst wall to anything other than mechanical breakage, the oocyst level, restricting access to template DNA (for most infecting species), and PCR interference by the surrounding fecal material have all hindered the use of PCR in Eimerian biology [11,14].

Although various PCR tests for identifying individual *Eimeria* spp. have been developed, relatively few studies have focused on the application of these methods for identifying *Eimeria* spp. in commercially farmed cattle around the world, particularly in Indonesia [10,12]. Using a multicopy genomic target and a nested PCR technique, the ITS-1 nested PCR test described by Lew *et al*. [16] and Kawahara *et al*. [11] identified more *Eimeria* spp. from more farms. The requirement of the nested assay for two PCR processes adds complexity, time, and cost, but the enhanced sensitivity was noticeable.

There have been only two reports of molecular studies of *Eimeria* spp. in cattle in Indonesia [10,12]. For the present study, a survey of bovine eimeriosis was conducted on Sulawesi Island, Indonesia. The multiplex PCR technique for *Eimeria* spp. identification was based on the ITS regions unique to each *Eimeria* spp. The use of nested multiplex PCR for the simultaneous detection of six species of *Eimeria* in cattle was first reported in Indonesia.

*E. bovis*, *E. zuernii*, *E. alabamensis*, *E. auburnensis*, *E. cylindrica*, and *E. ellipsoidalis* were identified by multiplex PCR using six ITS1-PCR primers. In comparison with tests that rely simply on the individual detection of each species, multiplex PCR procedures may be performed utilizing single-tube reactions, resulting in significant savings in time, labor, reagents, and experimental equipment [17].

In general, the sensitivity of multiplex PCR is not identical to that of individual PCR performed with each parasite’s DNA [18]. The detection sensitivity of multiplex PCR achieved in this study was the same as that of individual PCR (Figures-2 and 3). When the multiplex PCR assays described here are used, agarose gel electrophoresis of the second PCR products might yield all of the detection findings. This difference emphasizes the utility of genetic testing, and the multiplex PCR approach will be valuable in this regard.

Mixed *Eimeria* infections were more prevalent than single *Eimeria* infections. This test detects all six *Eimeria* spp. at the same time. Because this technique requires certain band profiles to overlap, species attribution might be challenging in mixed samples. Furthermore, because the amplification products are not quantitatively separated and seen on a gel, this approach does not distinguish between specific and non-specific amplicons. Multiplex PCR would minimize the requirement for a highly qualified specialist while also shortening diagnosis time by detecting six *Eimeria* spp. that are vital to cattle at the same time. If there were a well-designed multiplex PCR with high sensitivity and specificity, it would be a reliable diagnostic technique.

Thirty-six positive samples appeared to be mixed infections (100%) with more than 2 species present. In general, *E. bovis* and *E. zuernii* are regarded as extremely pathogenic. *E. zuernii, E. cylindrica*, *E. auburnensis*, *E. ellipsoidalis*, and *E. alabamensis* were identified in 47.2%, 19.4%, 27.8%, 44.4%, and 25% of samples, respectively (Table-2). Our findings are consistent with the previous study conducted in Indonesia, according to which, PCR analysis of *E. bovi*s was shown to be prevalent on Java Island [10] and Madura Island [12]. However, the PCR tests must be reviewed based on the purity of the sample, the number of oocysts in the DNA template, and the sequence of the particular primer to validate strains from diverse geographic origins and verify that they may be utilized globally.

For routine identification of *Eimeria* spp. in stool samples, the multiplex PCR test can be applied as a more sensitive alternative. This method takes less time and yields faster results when identifying various *Eimeria* spp. simultaneously [19].

## Conclusion

The nested multiplex PCR test in this study detected six pathogenic *Eimeria* spp. in beef cattle at once. The results of PCR optimization also depend on the DNA template, and it is recommended to use DNA from purified samples to minimize PCR barriers. In the future, a procedure to detect pathogenic *Eimeria* spp. in one tube reaction will offer economical and save diagnostic time. Thus, we can diagnose bovine eimeriosis quickly and apply early control techniques in the field.

## Authors’ Contributions

FE and MFN: Collection and analysis of data. FE and RWN: Data analysis and writing the manuscript. FE, RWN, DP, and JP: Research concept. All authors have read, revised, and approved the final manuscript.
